# Extracting antipsychotic polypharmacy data from electronic health records: developing and evaluating a novel process

**DOI:** 10.1186/s12888-015-0557-z

**Published:** 2015-07-22

**Authors:** Giouliana Kadra, Robert Stewart, Hitesh Shetty, Richard G. Jackson, Mark A. Greenwood, Angus Roberts, Chin-Kuo Chang, James H. MacCabe, Richard D. Hayes

**Affiliations:** 1 Department of Psychological Medicine, King’s College London, Institute of Psychiatry, Psychology, and Neuroscience, London, UK; 2 South London and Maudsley NHS Foundation Trust, London, UK; 3 Department of Computer Science, The University of Sheffield, Sheffield, UK; 4 Department of Psychosis Studies, King’s College London, Institute of Psychiatry, Psychology, and Neuroscience, London, UK

**Keywords:** Antipsychotic polypharmacy, Electronic health records, Precision, Recall

## Abstract

**Background:**

Antipsychotic prescription information is commonly derived from structured fields in clinical health records. However, utilising diverse and comprehensive sources of information is especially important when investigating less frequent patterns of medication prescribing such as antipsychotic polypharmacy (APP). This study describes and evaluates a novel method of extracting APP data from both structured and free-text fields in electronic health records (EHRs), and its use for research purposes.

**Methods:**

Using anonymised EHRs, we identified a cohort of patients with serious mental illness (SMI) who were treated in South London and Maudsley NHS Foundation Trust mental health care services between 1 January and 30 June 2012. Information about antipsychotic co-prescribing was extracted using a combination of natural language processing and a bespoke algorithm. The validity of the data derived through this process was assessed against a manually coded gold standard to establish precision and recall. Lastly, we estimated the prevalence and patterns of antipsychotic polypharmacy.

**Results:**

Individual instances of antipsychotic prescribing were detected with high precision (0.94 to 0.97) and moderate recall (0.57-0.77). We detected baseline APP (two or more antipsychotics prescribed in any 6-week window) with 0.92 precision and 0.74 recall and long-term APP (antipsychotic co-prescribing for 6 months) with 0.94 precision and 0.60 recall. Of the 7,201 SMI patients receiving active care during the observation period, 338 (4.7 %; 95 % CI 4.2-5.2) were identified as receiving long-term APP. Two second generation antipsychotics (64.8 %); and first -second generation antipsychotics were most commonly co-prescribed (32.5 %).

**Conclusions:**

These results suggest that this is a potentially practical tool for identifying polypharmacy from mental health EHRs on a large scale. Furthermore, extracted data can be used to allow researchers to characterize patterns of polypharmacy over time including different drug combinations, trends in polypharmacy prescribing, predictors of polypharmacy prescribing and the impact of polypharmacy on patient outcomes.

## Background

Clinical health records have been previously used to examine antipsychotic medication prescribing [1, 2]; however, the potential value of electronic health records (EHRs) remains underexplored. In the context of mental health care, EHRs contain large volumes of detailed information in free-text and structured fields, providing an important resource for conducting analyses using large samples and investigating a multitude of patient characteristics and outcomes simultaneously [3].

Studies investigating prescription databases [4–6] have been successful in deriving medication data for large populations and over long periods of time by predominately extracting data from structured fields (such as drop down menus, or dedicated response boxes) [6]. However, such studies have been restricted by the limited nature of the derived information [7]. Data on drug prescription, as well as related contextual information, is frequently embedded in free-text fields in mental health EHRs and this may be the only source of such information in the absence of e-prescribing or a Primary Care linkage. Traditionally, extracting free-text information has necessitated manual coding (where a researcher reads free-text and codes it by hand according to a defined set of coding rules) [8], which is time and labour intensive and therefore, not always feasible on a large scale. This can result in investigating a smaller than ideal sample [9–12]. EHR text has been analysed automatically using techniques such as natural language processing (NLP) for a variety of purposes [13]. However, although this has involved the identification of drugs [14], as far as we are aware, there have been no attempts to develop and validate techniques for characterising meta-data such as polypharmacy.

Automated extraction of information on medication prescribing is potentially valuable for investigating specific but important, clinical prescribing patterns such as the practice of prescribing more than one antipsychotic drug simultaneously, known as antipsychotic polypharmacy (APP), which may be challenging to identify through manual searches. The prevalence of APP in routine clinical practice has been estimated to vary between 10-30 % [15] in people with serious mental illness (SMI), despite little empirical evidence to support benefits associated with its use [16], and associations with adverse health outcomes, such as increased physical health problems (i.e. weight gain, diabetes, metabolic syndrome, dyslipidemia) and mortality [17–19]. We need to gain a better understanding of the clinical characteristics that predict APP prescribing and determine associated health outcomes. This information might be provided through research using the more “real-life” data present in EHRs. APP is thus an important exposure and potential confounder to be considered in studies investigating the impact of antipsychotic drugs in clinical settings and yet, as stated, is difficult to characterise on a large scale.

In this paper, we present and evaluate a novel process of extracting APP data from a large EHR data resource, utilising information available from both structured and free-text fields. In addition, we were able to use the processed data to estimate the prevalence of APP, as well as patterns in co-prescribing, for a six-month period in 2012.

## Methods

### Settings

South London and Maudsley NHS Foundation Trust (SLAM) is one of the largest providers of secondary healthcare in Europe, serving a geographic catchment of 1.23 million residents across four London boroughs (Lambeth, Southwark, Lewisham and Croydon) [20]. EHRs have been used by SLAM in all its services since 2006. In 2008 The Clinical Record Interactive Search (CRIS) system was developed [20], which allows researchers to search and retrieve anonymised SLAM EHRs, with over 230,000 cases currently represented in the system. CRIS was approval by the Oxfordshire Research Ethics Committee C (reference 08/H606/71).

### Sample

All adult service-users with a serious mental illness (SMI) diagnosis of schizophrenia (ICD-10: F20), schizoaffective disorder (F25) or bipolar disorder (F31) who received care from SLAM between January and June 2012 were considered. Diagnostic data were derived from free-text and structured fields within CRIS.

### Deriving antipsychotic polypharmacy data from EHRs

All antipsychotic drugs listed in the British National Formulary (BNF) 65 were considered. The BNF is a reference book containing information on pharmacology and prescribing of many medicines (including 29 antipsychotics) available on the British National Health Service (NHS). Structured fields for recording medications data are present in the source EHR interrogated by CRIS, and were used in this analysis, but these are infrequently completed. Information was also extracted from SLAM pharmacy records, although this only covers particular drugs that are subject to monitoring by the pharmacy such as clozapine. Most antipsychotic prescription information was extracted from free-text fields, including those recording clinician-patient encounters, and correspondence between healthcare professionals.

We extracted antipsychotic medication data from the free-text with a NLP information extraction application developed using General Architecture for Text Engineering (GATE) software [21], a suite of tools that facilitates the use and development of NLP applications and features. We applied NLP to extract a variety of grammatical features, which in turn were used to create specific filters to maximize precision and recall on instances of antipsychotic prescribing. For example, all instances of medication prescription that were not prescribed at the ‘present time’ (this refers to medication prescribed up until today, or from today with regard to the date the document was written) and that did not include a dose value were excluded at the point of data extraction. Therefore, any mentions of the drug without such supporting prescription information were not extracted, as these were deemed too imprecise.

#### APP algorithm

Long-term APP was defined as the concomitant use of two or more antipsychotics for six or more months. We considered that a concomitant use of antipsychotics for a six months duration reduces the possibility of misclassifying brief periods of co-prescribing during switching (a practice known as cross-titration, which typically takes up to 10 weeks [16, 22]) and ‘as required’ prescriptions as long-term APP; although, our approach cannot absolutely exclude cross-titration that has taken unusually long [16, 23].

**Fig. 1:**
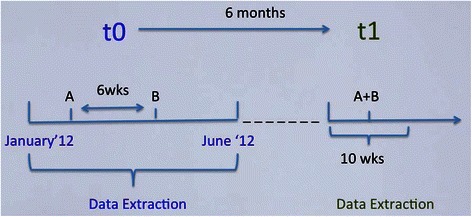
Antipsychotic polypharmacy algorithm

APP was ascertained using an algorithm comprising of two steps, as illustrated by Fig. 1. In step one, case records were examined to determine whether two or more antipsychotics were prescribed within a six-week period between January and June 2012. Co-prescribing at this stage (t0) was defined as baseline polypharmacy. At stage two, data on all patients with APP at baseline were re-examined six months after t0. A manual inspection of the data revealed that we were initially omitting outpatients who had less frequent clinical appointments and longer periods of time with no entry in the clinical record. Consequently, we specified that the follow-up search should begin at the point of first clinical event occurring six months or more after t0, which we designated as t1. Antipsychotic information was extracted from the clinical records, for the first ten weeks following t1, to determine whether the same set of antipsychotics were prescribed; if so, this was classified as ‘long-term’ polypharmacy.

During further development of the algorithm, we established that NLP-derived time and dose features were not sufficient to identify cases of APP, as they were not able to completely exclude historic medication information in clinical summaries, resulting in false positive instances (this refers to cases that are not true polypharmacy, but are detected as such by the application). Therefore, two additional filters were devised, applying the following exclusions: i) antipsychotic drugs with only a single annotation (by *annotation* we mean the identification and marking of spans of text that represent the prescribing of an antipsychotic) for the entire study period; and ii) antipsychotic drugs with multiple annotations but where all annotations were restricted to a single document for the entire study period. We reasoned that it was unlikely that a patient prescribed particular medication would have it mentioned in their notes only once over this period or only on a single day (i.e. a single document) over this period.

To evaluate the performance of the data extraction process (NLP application and APP algorithm), we measured two indicators of validity: precision and recall. Precision (equivalent to positive predictive value in psychometrics) represents the proportion of patients identified as polypharmacy considered to be ‘true positive’, out of all cases identified as such by the algorithm. Recall (equivalent to sensitivity) represents the proportion of patients on given medications who were identified as such by the algorithm.

#### Validation

Prior to testing the performance of the APP algorithm, we examined the NLP application on extracting information for specific antipsychotic agents prescribed at individual points in time (i.e. instances rather than episodes). The first author examined and manually coded free-text records over a 6-month period (January to June 2012) for a subset of 120 patients. We chose to examine six frequently prescribed antipsychotics [24] under the assumption that these medications would have a larger number of annotations for examination. Precision and recall for the extraction of clozapine prescriptions using this NLP algorithm is not included here as this has been described previously [25]. Consequently, the instances of antipsychotic prescribing varied from 328 to 1150 instances, by antipsychotic agent. We ran the NLP application over this set of unseen documents (that had not been used in the development of the NLP application) and compared the results to our manual coding of the same dataset.

**Fig. 2:**
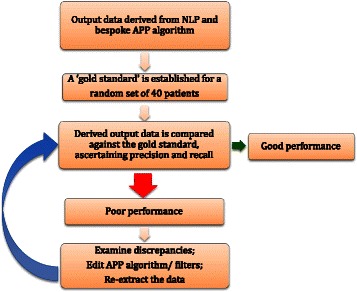
Antipsychotic polypharmacy validation process

As illustrated by Fig. 2, the final APP algorithm was derived following an iterative validation process. From those that were initially identified as being on polypharmacy by the application, we selected a random subset of 40 patients and manually coded their clinical records for APP, in order to ascertain its ‘true’ occurrence (also referred to as the ‘gold standard’). The extracted data were then compared against the gold standard to ascertain the validity of APP and to examine discrepancies. Instructions within the algorithm were then added or edited accordingly until a satisfactory performance was obtained. To confirm generalizability, the ‘final’ algorithm was tested out on a new subset of 30 randomly selected patients. To estimate recall, from all patients active in the observation period, we selected a random subset of 110 individuals.

### Analysis

Having assessed the precision and recall of the NLP application and APP algorithm, using the APP algorithm we estimated the prevalence of baseline and long term (≥6 months) APP. Prevalence estimates and 95 % confidence intervals were reported for baseline and long-term polypharmacy, as well as for long-term polypharmacy distribution by antipsychotic class and by individual agent.

## Results

As summarised in Table 1, the NLP application was able to identify individual instances of the selected antipsychotic agents with high precision, although recall levels were more modest. For the APP algorithm, the precision obtained from the final validation set of 30 patients was 0.92 for baseline and 0.94 for long-term APP. Recall was estimated at 0.74 and 0.60 for baseline and long- term APP respectively.

**Table 1:** Precision and recall per individual antipsychotic agent

Antipsychotic agent	N^a^	Precision (%)	Recall (%)
Amisulpride	619	97.4	61.0
Flupentixol	328	94.1	77.0
Haloperidol	747	94.0	57.0
Olanzapine	1150	95.0	69.3
Risperidone	737	95.0	64.1
Zuclopenthixol	390	97.0	67.5

^a^Number of annotations per antipsychotic

We determined that 7,201 adult patients with SMI diagnosis were active in SLAM services between January and June 2012. An estimated 830 (11.5 %; 95 % CI 10.8-12.3) patients were prescribed two or more antipsychotics in any six weeks between January and June 2012, and 338 (4.7 %; 95 % CI 4.2-5.2) were prescribed the same set of antipsychotics for six or more months.

Amongst patients prescribed long-term APP, co-prescribing two or more second-generation antipsychotics (SGAs) was most common (n = 219; 64.8 %; CI 95 % 59.7-69.9), followed by first generation (FGA) and SGA (n = 110; 32.5 %; CI 95 % 27.5-37.6) combinations, and two or more FGAs (n = 9; 2.7 %; CI 95 % 0.9-4.4).

Table 2 summarises long-term co-administration patterns by individual agents. Similarly to co-administration by class, the combination of two (or more) first generation antipsychotics (FGAs) was relatively rare. The most common antipsychotic used in combination was clozapine, combined with at least one other SGA.

**Table 2:** Prevalence of long-term antipsychotic polypharmacy combinations (n = 338)

Antipsychotic medication	n	Plus at least one other FGA^a^ n (%)	Plus at least one other SGA^a^ n (%)
First Generation Antipsychotics (FGA)^a^			
Chlorpromazine	8	1 (12.5)	7 (87.5)
Flupentixol	26	6 (23.1)	20 (76.9)
Fluphenazine	4	2 (50.0)	2 (50.0)
Haloperidol	30	5 (16.7)	25 (83.3)
Levomepromazine	1	-	1 (100.0)
Pericyazine	1	1 (100.0)	-
Pimozide	2	-	2 (100.0)
Pipothiazine	10	2 (20.0)	8 (80.0)
Sulpride	33	1 (3.0)	32 (97.0)
Trifluoperazine	3	-	3 (100.0)
Zuclopenthixol	25	-	25 (100.0)
Second Generation Antipsychotics (SGA)^a^			
Amisulpride	118	18 (15.3)	100 (84.7)
Aripiprazole	79	12 (15.2)	67 (84.8)
Clozapine	168	27 (16.1)	141 (83.9)
Olanzapine	95	44 (46.3)	51 (53.7)
Paliperidone	40	8 (20.0)	32 (80.0)
Quetiapine	21	11 (52.4)	10 (47.6)
Risperidone	64	21 (32.8)	43 (67.2)

^a^These are overlapping categories; antipsychotic combinations may include additional FGAs or SGA where patients are prescribed more than 2 antipsychotics simultaneously

## Discussion

To our knowledge, this is the first report investigating the feasibility and yield for a process of extracting APP data from both structured and free-text fields in EHRs, using a combination of NLP and a bespoke algorithm. This process enabled us to identify instances where specific antipsychotic agents were prescribed, then classify baseline and long term APP profiles over time.

The NLP application combined with the APP algorithm performed at a high precision, suggesting that individuals classified as being prescribed APP were very likely to be classified correctly. The moderate recall suggested that we were less able to detect all APP cases. In designing the APP algorithm, we noticed that some of the rules used to decrease the false positive cases of APP, filtered out some of the ‘true’ APP cases, requiring a trade-off decision. Although detecting all cases is desirable, especially when investigating relatively uncommon phenomenon such as polypharmacy, we chose to prioritise precision over recall due to the large number of non-cases in the sample, which might be expected to dilute the impact of any such misclassification in future analyses. Similarly, the NLP application was developed to favour precision over recall. In this study we considered date specific recall when evaluating the NLP application for extracting individual medications; however, in longitudinal studies a single patient often has a number of documents containing the same prescription information, therefore relatively low recall could be compensated by combining results extracted from several documents.

We estimated that just under five percent of all adult patients with SMI were prescribed two or more antipsychotics for six or more months. Although this is comparable to some research investigating APP with longer duration (Morrato et al. [26] found 6.4 % APP prevalence in Medicaid population), it is somewhat lower in comparison to other previous research (10-30 %) [15]. The lower prevalence could be attributable to a more conservative approach that was adopted in detecting APP, by examining long-term co-prescription with a minimum duration of six months. Some previous studies that have examined concomitant prescribing for 28 days [27], 6 weeks [28, 29] and 60 days [4, 30] may have included instances of ‘as required’ medication and switching. It is also possible that some polypharmacy cases were omitted because we prioritized precision over recall in developing the NLP application and algorithm. On the other hand, our findings are consistent with previous research on antipsychotic co-administration, where two or more SGAs, and FGA-SGA combinations are found to be the most prevalent combinations in clinical settings [15, 28, 29, 31, 32].

Previous research has suggested that olanzapine and risperidone are most commonly combined in co-prescribing [28, 33], whereas clozapine was the most commonly co-prescribed antipsychotic in our sample. Although the therapeutic benefits of clozapine co-prescribing has been previously called into question [34], this antipsychotic remains one of few that has some empirical support when used in polypharmacy [35]. Furthermore, most research to date has examined shorter periods of APP (i.e. 6 weeks) [28], whereas studies investigating long-term APP have reported a higher prevalence of clozapine as a component [4]. Clinically, this may indicate that patients persistently prescribed APP over longer periods of time are different from those on other forms of APP (i.e. short bouts of co-prescribing); more specifically, it is likely that this sub-group are more unwell and possibly treatment refractory [36].

Our process of extracting medication data from EHRs has a number of advantages. For example, in instances where structured fields are poorly populated or incomplete, using supplementary information available in freetext fields provides more detailed and complete information of treatments. A particular advantage of NLP is its ability to take into account the linguistic context around terminology of interest. Therefore, we were able to identify and exclude negation statements, past rather than current prescribing, speculations about future prescribing and instances in the text where the drug is mentioned as being taken by a person other than the patient. Furthermore, the APP algorithm allowed us to distinguish between different modes of polypharmacy administration, such as shorter (which would potentially include ‘as required’ and switching occurrences) and longer forms of co-prescribing.

Data from EHRs are a source of rich and diverse contextual information, much of which may be embedded in free-text fields. The process described here, may be adapted to extract an array of factors, which may predict antipsychotic polypharmacy and/or confound associations between APP and mental or physical health outcomes. Routinely collected EHRs capture a range of populations, such as patients in different clinical settings (i.e. inpatients/outpatients) and with different socio-demographic profiles who have been previously been under-represented and/or under-investigated in research. Moreover, EHRs more closely approximate real-life clinical practice than formal research projects involving *de novo* data collection, permitting the identification of trends in medication prescribing that are not otherwise captured by clinical trials. This could be valuable information that can be fed back into prescribing guidelines. Finally, the historic nature of EHRs allows longitudinal research, where medication profiles can be examined in relation to multiple predictors and outcomes.

Our current protocol for extracted APP data has a number of limitations, which should be borne in mind. As indicated by the recall for individual antipsychotics and long-term antipsychotic polypharmacy, our approach may under-estimate the true prevalence of APP. Furthermore, the output data depends on the quality and accuracy of clinical entries [20], which may vary by clinicians and services. Finally, it is important to note that we examined antipsychotic polypharmacy over a relatively short period of time, and it is possible that our data reflects a specific pattern in medication prescribing during that period.

## Conclusions

We have developed a novel process for extracting APP information from mental health electronic patient records. We have demonstrated that the combination of natural language processing and a bespoke algorithm can be an effective approach to extracting APP data. We were able to detect APP with high precision and modest recall. Once extracted these data can be used to allow researchers to characterize patterns of polypharmacy over time including different drug combinations, trends in polypharmacy prescribing, predictors of polypharmacy prescribing and the impact of polypharmacy on patient outcomes (such as mortality and physical health consequences). The use of NLP combined with a bespoke algorithm is likely to be applicable to similarly structured clinical datasets where medications data is held in free-text. Essentially we have provided an example of an approach which other researchers may trial in their own datasets with some modification to suit their specific needs and source data.

## Abbreviations

APP: Antipsychotic polypharmacy
EHRs: Electronic health records
SLAM: South London and Maudsley
SMI: Serious mental illness
NLP: Natural language processing
CRIS: The Clinical Record Interactive System
BNF: British National Formulary
